# Smoking Determinants in Turkish University Students

**DOI:** 10.3390/ijerph6082248

**Published:** 2009-08-12

**Authors:** Feryal C. Celikel, Serhat Celikel, Unal Erkorkmaz

**Affiliations:** 1 Gaziosmanpasa University School of Medicine, Department of Psychiatry, Tokat, Turkey; 2 Gaziosmanpasa University School of Medicine, Department of Chest Diseases, Tokat, Turkey; E-Mail: scelikel@gmail.com; 3 Gaziosmanpasa University School of Medicine, Department of Biostatistics, Tokat, Turkey; E-Mail: uerkorkmaz@gmail.com

**Keywords:** smoking, emotional correlates, university students, Turkey, nonclinical

## Abstract

The aim was to explore the prevalence and the correlates of smoking in a group of Turkish university students. A sample of 1,870 students (21.2 ± 2.0 years old) completed the Beck Depression Inventory, Beck Hopelessness Scale, Anxiety Sensitivity Index, 20-item Toronto Alexithymia Scale. Smoking was highly prevalent (35.9%) in this sample. Male gender (*OR* = 2.72, CI 2.15–3.44), and parental smoking (*OR* = 1.41, CI 1.13–1.78) were factors associated with increased likelihood of smoking. Higher depressive symptoms and hopelessness levels were significantly related to smoking behavior. Smoking behavior might initiate as a mild and transient habit and unfortunately could become more serious and lead to an actual dependence. The results of this study show that it is necessary to pay attention to levels of depression and hopelessness, as well as parental influence.

## 1. Introduction

The prevalence of smoking still remains high among young adults, with the age of onset actually declining over time [1,2]. Numerous studies showed that an increasing proportion of Turkish youth smoke tobacco [3–5]. The prevalence of smoking ranges between 30% and 63% [5–7]. There are reports suggesting that smoking rate tends to increase in the universities [5,8]. Aslan *et al.* [9] found that the smoking prevalence among final year university students was significantly higher than the first year students. In a study among medical students, one-third of non-smokers in the first year had become smokers by the end of the sixth year [8].

Considerable research about smoking has focused on psychological predictors of the onset or initiation of smoking behavior. The emotional, social, psychological, and behavioral factors are considered as factors affecting the smoking behavior in young individuals [10]. Several studies from Turkey had previously reported a male predominance over females [3–5]. Despite considerable research examining the association between parental socioeconomic level and adolescent smoking, the nature of the association is still unresolved. There are studies which stated that youths who had more spending money were better able to afford tobacco and were more likely to smoke cigarettes [11,12]. After reviewing 21 such prospective studies, it was concluded that an inverse association between economic difficulties and adolescent smoking was supported by 76% of the studies [13]. In a review article, parental socioeconomic level and adolescent smoking were reported to be inversely associated [14].

Depressive symptoms seem to be a significant risk factor for increased cigarette smoking in college students [15,16]. Smokers, who are depressed tend to smoke more cigarettes than non-depressed individuals who smoke [17]. In addition, depressed individuals are less successful in their efforts to stop smoking [18]. Ingesting nicotine through smoking has been thought to be an attempt to self-medicate one’s negative affective states [19]. Negative affect and feelings of hopelessness have also been found to be integral in the maintenance of smoking [20]. Smoking may be a coping mechanism for dealing with boredom and frustration or a way to reduce stress or maintain personal energy.

There are studies which suggest an association between anxiety sensitivity (AS) and smoking [21,22]. AS is the fear of anxiety-related sensations. It arises from beliefs that anxiety symptoms lead to disastrous physical or emotional effects [23]. Thus, AS is theorized to predispose individuals to the development of elevated levels of anxiety. The findings suggest that individuals start smoking because of problems with emotional regulation [24]. Alexithymia was originally characterized as an inability to find words to describe one’s feelings but is also conceptualized as a dysfunction in identifying (or awareness to) one’s feelings [25]. Because of their limitations in regulating emotions, alexithymic subjects may try to regulate their uncontrollable sensations by maladaptive self-stimulatory behaviors, such as smoking.

Family-related variables have also been associated with smoking in young adults. Adolescents are at substantially higher risk of smoking if at least one of their parents smokes [26]. The exact nature of the relationship is unclear; while genetics may contribute to this link, modeling most likely plays a large part in this connection [17,20]. Given the growing concern about increased smoking in the young, further understanding of the motivation for nicotine use is clearly warranted. With the majority of smokers beginning to smoke by adolescence, young individuals with emotional dysfunctions are expected to have a higher risk for smoking compared with those without such problems [27,28]. These findings point out the importance of counseling during adolescence as well as university years. The aim of this research was to explore the prevalence and the correlates of smoking behaviour in a group of Turkish university students, and to identify a possible correlation between smoking habits and sociodemographic variables, current hopelessness, depression levels, anxiety proneness, and alexithymia in this group of Turkish university student smokers.

## 2. Methods

The study was conducted on students attending 24 departments of six faculties at Gaziosmanpasa University in Tokat, Turkey. From the total of 7,964 students (3,020 females and 4,944 males) studying at this University, 2,168 subjects were selected with randomized stratified sampling. None of the students declined participation. Two-hundred ninety-eight students were excluded because of incomplete and contradictory answers to the smoking questions. Therefore, a total of 1,870 students were enrolled. Of the sample, 52.2% were males and the mean age was 21.2 ± 2.0.

The current study was approved by the local ethics committee. After the description of the study, all subjects gave written consent and completed the questionnaires during school time after explanations and assurances of confidentiality. Anonymity was strictly maintained. A set of self-report questionnaires was given to all subjects and collected immediately afterward.

In our questionnaire, in order to define the smoking status of the students, two questions were asked: 1. “Have you smoked 100 cigarettes in your lifetime?” 2. “How do you smoke now?”. The respondents, who answered the first question as “yes” and answered the second question as “everyday or occasionally”, were defined as current smokers. The respondents, who answered the first question as “no”, were nonsmokers. Those, who answered the first question as “yes” but answered the second question as “none”, were former smokers.

The 21-item Beck Depression Inventory (BDI) [29] was used to assess depression. The BDI is a self-report questionnaire. The 21 items correspond to symptoms such as mood, pessimism, and suicidal ideas. Subjects rate each item on a four-point Likert scale from 0 (absent) to 3 (severe). Higher scores indicate greater depression. The BDI is an internally consistent and valid measurement. A valid and reliable Turkish translation of the scale was used [30].

The level of hopelessness was assessed by using the Beck Hopelessness Scale (BHS), which is a 20-item, self-administered rating scale designed to measure the negative expectancies of individuals concerning themselves and their future life [31]. The total score ranges from 0 to 20, and the level of hopelessness increases with increasing scores. According to the original cut-off points, scores of 0–3 indicate no hopelessness, 4–8 mild hopelessness, 9–14 moderate hopelessness, and scores of 15–20 indicate severe hopelessness. In this study, a BHS score ≥9 was chosen to indicate hopelessness. A valid and reliable Turkish translation of the scale was used [32,33].

Alexithymia was assessed with the self-report 20-item Toronto Alexithymia Scale (TAS-20), which is comprised of 20 items rated on 5-point Likert scales ranging from 1 (strongly disagree) to 5 (strongly agree) [34,35]. Total scores range from 20 to 100. A cut-off score of 61 was used to define alexithymia as recommended. High values correspond to problems in emotional functioning. The valid and reliable Turkish translation was used [36].

Participants completed the 16-item Anxiety Sensitivity Index (ASI) [37] to assess second order anxiety, defined as fear of anxiety-related sensations. The ASI measures the degree to which participants fear negative consequences stemming from anxiety symptoms. Respondents indicated the degree to which individual items characterized them on a 5-point Likert scale ranging from 0 (very little) to 4 (very much). ASI has also been found valid in Turkish population studies [38].

### 2.1. Statistical Analysis

Chi-square test was used to compare the categorical variables between the smoker and non-smoker groups. The variable distribution was not normal according to a Kolmogorov-Smirnov normality test, so a Mann-Whitney U test was used to compare the categorical variables between the groups. Spearman Correlation Analysis was used to explore the relation between smoking behavior and possible risk factors. Multiple Logistic Regression Analysis was used to determine the effect of the risk factors on smoking behavior. The continuous variables were presented as the mean ± standard deviation (SD). The categorical variables were presented as a count and percentage. A *p*-value < 0.05 was considered as significant. Analyses were performed using the commercial software SPSS 15.0, (Chicago, IL).

## 3. Results

Of the total sample (n = 1,870), 35.9% were current smokers. Table 1 shows the distribution of the characteristics of the smoker and the non-smoker group of students. Males smoked significantly more (46.2%) than females (24.4%). Students, whose parents smoked (39.6%), were smoking significantly more than those with no parental smoking (32.8%). Males and females did not differ with regard to parental smoking (50.6% vs. 54.3%, respectively; *χ^2^* = 2.482, *p* = 0.12). In the total sample, the mean age of starting smoking was 16.0 ± 2.8 years. Males started smoking earlier (15.7 ± 3.0) than females (16.9 ± 2.3) (*Z* = 5.325, p < 0.001). Of the smokers, 21.7% (n = 146) smoked for the first time in their university years.

Table 2 shows comparison of the mean scores of the measures between the two groups. BDI and BHS scores were significantly related to smoking behavior (*p* < 0.001 and *p* = 0.01, respectively). Cohen’s d values suggested weak associations between these variables (BDI and BHS) and smoking.

Table 3 shows the results of the Logistic Regression Analyses in determining the risk factors of smoking behavior. Male gender (*OR* = 2.72, CI 2.15–3.44) and parental smoking (*OR* = 1.41, CI 1.13–1.78) were factors associated with an increased likelihood of smoking. Higher BDI (*OR* = 1.23, CI 1.13–1.33) and lower TAS-20 (*OR* = 0.83, CI 0.74–0.93) scores were related to increased risk.

## 4. Discussion

Our findings revealed that 35.9% of participating university students were smokers. This rate is consistent with the results of previous studies [5–7,39,40]. Because our study was conducted in one college only, the results may only represent the frequency of smoking and its risk factors among college students in a semi-rural area in Turkey. A number of demographic, school and parent related variables were found to be associated with smoking in our sample of university students. In determining the risk factors of smoking behavior, male gender and parental smoking were factors associated with an increased risk of smoking.

Male students smoked more (46.2%) than their female counterparts (24.4%), and had 2.72 times higher risk of smoking compared to females. This is in line with several studies from Turkey which reported a male predominance over females [3,4,41,42]. In line with previous research [26], our results indicated that parental smoking is influential in determining the likelihood of smoking in young adults. In our sample, participants were more likely to smoke if their parents smoked. Antismoking campaigns may need to target parents to help them understand their influence.

In our study, socioeconomic conditions did not seem to play a role in smoking. There are studies with adequate sample sizes which have failed to find a relationship between a measure of socioeconomic status. In order to assess socioeconomic position, studies generally used various measures, such as educational levels or occupation of parents. In our study, we asked the students whether or not they had difficulty in affording school expenses. The failure to find a relationship between smoking and socioeconomic status may be explained by our sampling, which is solely from a rather homogenous socioeconomic group.

In our sample, the mean age of starting smoking was around 16 years; boys started smoking earlier (around 15 and 16 years) than girls (around 16 and 17 years). One fifth of the smokers reported smoking for the first time in their university years.

In the present study, higher depressive symptom levels, as measured by the BDI, were risk factors in the prediction of smoking behavior. This finding is consistent with previous research [20,26,43]. Anda *et al.* [44] have shown that young adult smokers were significantly more depressed than nonsmokers, even when matched for adverse life events. Although in our sample hopelessness levels were found to be significantly higher in the smoker group compared to nonsmokers, this effect has gone away when controlling for other factors and demographic effects.

Our results did not support the hypothesis that high anxiety increases the risk of having a smoking habit [21,45]. The failure to find such a relationship may be explained by our method. We used ASI as parameter of anxiety sensitivity in this study. This allowed us to detect “fear of anxiety-related sensations”, but not “anxiety disorder”. Prospective analyses might reveal the specific relationship between anxiety and tobacco use, if individuals with prior high anxiety sensitivity tend to smoke more. Another concern was that our sample reflected a relatively limited range of anxiety sensitivity scores. Finally, lower TAS-20 scores were related to increased risk in our sample of university students. Thus, the notion that individuals with specific deficits smoke for purposes of self-medication [46] was not supported. Our findings suggest that the affect regulation deficits in alexithymia may play a protective role in the onset or the maintenance of smoking behavior.

Our study was subject to certain restrictions. First, the present study was cross-sectional and not longitudinal. Second, all evaluations were based on self-report measures. The use of a self-report measure may be unable to investigate the lack of awareness of feelings. Alexithymic subjects were perhaps unable to express themselves correctly because of their difficulties in cognitive processing of emotions. It might be better to use more objective measures of psychological status (e.g., standardized interviews) or smoking status. Third, the findings of this study on predominantly young adults of mean age around 21 years might not apply to persons outside this age range. The subjects were only university students, and were selected from a socially, economically, and educationally homogeneous population. Our sample represents a well-educated and middle to upper class university population, limiting the generalizability of the findings.

Although the present study does not shed light on the causal nature of the relationship between smoking and depressive symptoms, it supports a relationship between smoking habit and hopelessness and depressive symptom levels. The sample size was large enough to detect possible associations between the variables examined. These findings add to accumulating evidence that depressive symptoms are a risk factor for increased cigarette smoking in college students. In spite of the large body of research in Western societies on the frequencies and correlates of smoking behavior, there are still few studies in developing societies and in the rural areas of these societies. Our results demonstrate that smoking is a major concern among young adults of these societies as well.

## 5. Conclusions

In the present study, it appears that male gender, parental smoking, higher depression and hopelessness levels were the best predictors of smoking behavior. Current cigarette consumption was not related to anxiety sensitivity or alexithymia in this group of university students. Smoking was highly prevalent (35.9%) among the students of Tokat, Gaziosmanpasa University, as it is throughout Turkey. Twenty-one per cent of the smokers started smoking in their university years; thus, universities are important settings for campaigns against smoking. Smoking behavior might initiate as a mild and transient habit and unfortunately could become more serious and lead to an actual dependence. The results of this study show that it is necessary to pay attention to levels of depression and hopelessness, as well as parental influence.

## Figures and Tables

**Table 1.** Distribution of the characteristics of the smoker and the non-smoker groups.

	**Smoker**	**Non-Smoker**	***χ*^*2*^**	***p***

	**n (%)**	**n (%)**		

**Gender**				
male	456 (46.2)	530 (53.8)	96.334	**<0.001**
female	216 (24.4)	668 (75.6)		

**Student lives**				
with family	93 (31.0)	207 (69.0)	3.702	0.05
away from family	574 (36.8)	985 (63.2)		

**Student has**				
no difficulty in affording school expenses	205 (34.1)	397 (65.9)	1.737	0.19
has difficulty in affording school expenses	462 (37.2)	780 (62.8)		

**Parental smoking**				
parents smoke	381 (39.6)	582 (60.4)	9.174	**<0.01**
parents don’t smoke	287 (32.8)	589 (67.2)		

**Table 2.** Comparison of BDI, BHS, TAS-20, and ASI scores between the smoker and the non-smoker groups.

		**Smoker**		**Non-Smoker**				

**Test**	**n**	**mean ± SD**	**N**	**mean ± SD**	***Z***	***p***	**Cohen’s d**	**Effect size rho**

**BDI**	614	16.74 ± 8.72	1126	15.05 ± 7.81	3.799	**<0.001**	**0.20**	**0.10**
**BHS**	598	5.37 ± 4.36	1130	4.79 ± 4.02	2.459	**0.01**	**0.13**	**0.06**
**TAS-20**	624	51.34 ± 10.38	1143	51.34 ± 9.68	0.146	0.88	0	0
**ASI**	613	25.69 ± 11.84	1142	25.79 ± 11.65	0.141	0.89	−0.008	−0.004

BDI: Beck Depression Inventory; BHS: Beck Hopelessness Scale; TAS-20: Toronto Alexithymia Scale; ASI: Anxiety Sensitivity Index.

**Table 3.** Risk factors of smoking behavior.

**Risk Factors**	***n***	***β***	***p***	***OR***	**95.0% CI for OR**

**Gender**					
Female *	709			1	
male	767	1.001	**<0.001**	2.72	2.15–3.44

**Student lives**					
with family *	235			1	
away from family	1241	0.206	0.21	1.22	0.89–1.70

**Student has**					
no difficulty in affording school expenses*	494			1	
has difficulty in affording school expenses	982	0.011	0.93	1.01	0.79–1.30

**Parental smoking**					
parents don’t smoke *	774			1	
parents smoke	702	0.346	**<0.01**	1.41	1.13–1.78

Depressive symptom level (BDI) /SD**	1476	0.025	**<0.01**	1.23	1.13–1.33
Hopelessness level (BHS) /SD **	1476	0.002	0.92	1.01	0.88–1.15
Alexithymia level (TAS-20)/ SD**	1476	−0.019	**<0.01**	0.83	0.74–0.93
Anxiety sensitivity level (ASI) /SD **	1476	0.001	0.95	1.01	0.90–1.14

Dependent variable is smoking behavior.

BDI: Beck Depression Inventory; BHS: Beck Hopelessness Scale; TAS-20: Toronto Alexithymia Scale; ASI: Anxiety Sensitivity Index.

*: This category is the reference category.

**: OR’s are expressed as the change for one SD. SDs are for BDI: 8.18; BHS: 4.14; TAS-20: 9.93 and ASI:11.71
